# Beta-blockeRs tO patieNts with CHronIc Obstructive puLmonary diseasE (BRONCHIOLE) – Study protocol from a randomized controlled trial

**DOI:** 10.1186/s13063-019-3907-1

**Published:** 2020-01-30

**Authors:** Josefin Sundh, Anders Magnuson, Scott Montgomery, Pontus Andell, Gustaf Rindler, Ole Fröbert, the BRONCHIOLE investigators, Malgorzata Przybyszewska, Anders Blomberg, Magnus Widmark, Andreas Palm, Wolfgang Greger, Jens Ellingsen, Lennart Råhlén, Taivo Kipper, Mikael Hasselgren, Carolina Smith, Florim Delijaj, Kaj Possler, Johanna Nilsson, Niklas Stenersen, Han Nguyen, Dan Curiac, Lowie E. G. W. Vanfleteren, Lars Johansson, Folke Sjöberg, Magnus Ekström, Johan Sanmartin Berglund, Anders Lökke

**Affiliations:** 1 Department of Respiratory Medicine, Faculty of Medicine and Health, Örebro University, Örebro, Sweden; 2 Clinical Epidemiology and Biostatistics, Örebro University, Örebro, Sweden; 3 Clinical Epidemiology Division, Department of Medicine, Karolinska Institutet, Solna, Stockholm Sweden; 4 Department of Epidemiology and Public Health, University College London, London, UK; 5 Unit of Cardiology, Department of Medicine, Karolinska Institutet, Stockholm, Sweden; 6 Heart and Vascular Division, Karolinska University Hospital, Stockholm, Sweden; 7 Lekeberg Primary Health Care Centre, Örebro, Sweden; 8 Department of Cardiology, Faculty of Medicine and Health, Örebro University, Örebro, Sweden

**Keywords:** Beta-blocker, Cardiovascular event, COPD, Exacerbation, Mortality, Pragmatic randomized trial, Real-world evidence

## Abstract

**Background:**

Observational studies indicate that beta-blockers are associated with a reduced risk of exacerbation and mortality in patients with chronic obstructive pulmonary disease (COPD) even without overt cardiovascular disease, but data from randomized controlled trials (RCT) are lacking. The aim of this RCT is to investigate whether beta-blocker therapy in patients with COPD without diagnosed cardiovascular disease is associated with a decreased 1-year risk of the composite endpoint of death, exacerbations, or cardiovascular events.

**Methods:**

The Beta-blockeRs tO patieNts with CHronIc Obstructive puLmonary diseasE (BRONCHIOLE) study is an open-label, multicentre, prospective RCT. A total of 1700 patients with COPD will be randomly assigned to either standard COPD care and metoprolol at a target dose of 100 mg per day or to standard COPD care only. The primary endpoint is a composite of death, COPD exacerbations, and cardiovascular events. Major exclusion criteria are ischemic heart disease, left-sided heart failure, cerebrovascular disease, critical limb ischemia, and atrial fibrillation/flutter. Study visits are an inclusion visit, a metoprolol titration visit at 1 month, follow-up by telephone at 6 months, and a final study visit after 1 year. Outcome data are obtained from medical history and record review during study visits, as well as from national registries.

**Discussion:**

BRONCHIOLE is a pragmatic randomized trial addressing the potential of beta-blockers in patients with COPD. The trial is expected to provide relevant clinical data on the efficacy of this treatment on patient-related outcomes in patients with COPD.

**Trial registration:**

ClinicalTrials.gov, ID: NCT03566667. Registered on 25 June 2018.

## Background

Chronic obstructive pulmonary disease (COPD) is associated with a high risk of exacerbation and mortality [1, 2] and of comorbid conditions such as cerebrovascular disease, ischemic heart disease, heart failure, and atrial fibrillation [3]. Cardiac disease is the most frequent cause of death in patients with COPD [4].

Treatment with cardio-selective beta-1-antagonists (beta-blockers) has been demonstrated to reduce mortality in patients with COPD and concomitant heart failure or previous myocardial infarction [5, 6]. The medication is confirmed to be safe, with no associated dyspnea or significant adverse effect on lung function [7]. Observational studies also suggest that beta-blockers reduce COPD exacerbation frequency and increase survival, independent of overt heart disease [8–10].

Multiple mechanisms might explain the beneficial effects of beta-blockers in patients with COPD without overt heart disease, including the possibility of a primary preventive effect countering the increased risk of incident cardiovascular disease in COPD. Cardiovascular disease is highly prevalent, but largely underdiagnosed in COPD [11], and the additional trauma of a COPD exacerbation could trigger and expose underlying but previously undiagnosed cardiac dysfunction [12]. A recently published Danish observational study showed reduced hospitalization and mortality of COPD patients treated with beta-blockers compared with those receiving other antihypertensive drugs [13]. Heart rate is also a suggested target for intervention, as it is generally increased and associated with higher all-cause and cardiovascular mortality in all stages of COPD [14]. Patients with COPD may exhibit secondary pulmonary hypertension and right ventricular failure leading to decreased cardiac output despite preserved left ventricular function [15], which could be counteracted by beta-blockers [16]. Many patients with COPD use maintenance treatment with inhaled beta-2-agonists, which might affect the beta-1 pathway and aggravate tachycardia and sympathetic stress and also lead to down-regulation of beta-2-receptors. Beta-1 blockers may compensate, at least partly, for both effects [17]. It has also been suggested that beta-blockers may prevent COPD exacerbations by reducing systemic inflammation [18] and by decreasing the release of the broncho-constrictor endothelin [19].

Although there is clear evidence from randomized controlled trials (RCT) for use of beta-blockers in patients with COPD and concomitant heart disease [5, 6], to our knowledge no RCTs have reported an effect of beta-blocker therapy in patients with COPD without heart disease. This paper presents the protocol for a Swedish national multicentre pragmatic RCT, Beta-blockeRs tO patieNts with CHronIc Obstructive pulmonary diseasE (BRONCHIOLE), with the primary aim of investigating whether beta-blocker therapy in patients with COPD and no overt cardiovascular disease protects against death, COPD exacerbations, and cardiovascular events.

## Methods

### Study design

BRONCHIOLE is a pragmatic RCT, which is defined as a study characterized by minimal inclusion and exclusion criteria, a low number of follow-up visits with low complexity, and an intervention that is additional to standard care but with no placebo control [20]. Pragmatic trials integrate randomized treatment within the usual care system via the collection of baseline and outcome data from health records and registries rather than by study-specific visits [20, 21]. The (PRagmatic Explanatory Continuum Indicator Summary − 2 (PRECIS-2) tool was developed to support the explicit matching of design decisions to how the trial results are intended to be used [22]. It includes nine domains scoring from 1 (very explanatory) to 5 (very pragmatic); Eligibility criteria (Who is selected to participate in the trial?), Recruitment (How are participants recruited in the trial?), Setting (Where is trial being done?), Organization (What expertise and resources are needed to deliver the intervention?), Flexibility/delivery (How should the intervention be delivered?), Flexibility/adherence (What measures are in place to ensure participants adhere to the intervention?), Follow-up (How closely are participants followed up?), Primary outcome (How relevant is it to participants?), and Primary analysis (To what extent are all data included?). We found that our study design corresponded to the highest score on all items except one. The reduced the score on the recruitment item was because we mainly recruited from clinical care but added advertising campaigns in order to recruit more people. However, as we found our total score to be 24 out of 25, we believe that BRONCHIOLE can be categorized as an effective pragmatic trial.

### Objectives

The primary objective of BRONCHIOLE is to investigate whether the addition of 100 mg per day of metoprolol to standard COPD care is associated with a decreased rate of a composite measure of death, COPD exacerbations, or cardiovascular events at 1year. The secondary objective is to ascertain effects on each of the primary outcome components (Table 1).

**Table 1:** Objectives

Primary objective	
• Does metoprolol therapy up to 100 mg per day in addition to standard care reduce the rate of the composite measure of death, COPD exacerbations, or cardiovascular events at 1 year in patients with COPD and no diagnosed cardiovascular disease at baseline, compared with standard care only?	
Secondary objectives	
• Does treatment with metoprolol at a target dose of 100 mg in addition to standard care reduce the 1-year risk of death (all-cause and cause-specific) in patients with COPD and no diagnosed cardiovascular disease at baseline, compared with standard care only?	
• Does treatment with metoprolol at a target dose of 100 mg in addition to standard care reduce the 1-year risk of cardiovascular events in patients with COPD and no diagnosed cardiovascular disease at baseline, compared with standard care only?	
• Does treatment with metoprolol at a target dose of 100 mg in addition to standard care reduce the 1-year risk of COPD exacerbations in patients with COPD and no diagnosed cardiovascular disease at baseline, compared with standard care only?	

Primary and secondary objectives of the BRONCHIOLE trial. *COPD* Chronic obstructive pulmonary disease

### Recruitment and study population

Patients with a physician diagnosis of COPD managed in primary or secondary care settings are recruited using invitation letters, notices in local newspapers and social media, and invitation during clinical practice visits. Candidate participants will receive oral and written information about the study and sufficient time to read and consider the information contained and decide whether to participate in the study. The participants will be provided opportunities to ask questions during screening phone calls and at the inclusion visit. Patients will enter the study after signing the informed consent form in the presence of the investigator. The inclusion and exclusion criteria are summarized in Table 2.

**Table 2:** Inclusion and exclusion criteria

Inclusion criteria	
• A diagnosis of COPD confirmed by spirometry showing a post-bronchodilator value of FEV_1_/FVC < 70 and a history of tobacco smoking or occupational/environmental exposure to smoke, gas, or dust	
• ≥40 years of age	
• Sinus rhythm ≥50/min and < 120/min at inclusion	
• Written informed consent	
Exclusion criteria	
• Known hypersensitivity to metoprolol or related derivatives	
• AV block II or III unless treated with a pacemaker	
• Sinus bradycardia (resting heart rate < 50/min)	
• Sick sinus syndrome unless treated with a pacemaker	
• Atrial fibrillation/flutter	
• Clinical signs or previous diagnosis of heart failure, angina pectoris, myocardial infarction, cerebrovascular disease, or critical peripheral ischemia	
• Systolic blood pressure < 90 mmHg	
• Tachyarrhythmia other than sinus tachycardia	
• Sinus tachycardia > 120/min	
• Severe bronchial asthma	
• Current beta-blocker treatment	
• Inability to provide informed consent	
• Age < 40 years	
• Acute on-going exacerbation of COPD	
• Previous randomization in the trial	
• Pregnancy (confirmed by pregnancy test when appropriate)	

Inclusion and exclusion criteria of the BRONCHIOLE trial*. COPD* Chronic obstructive pulmonary disease, *FEV*_*1*_ Forced expiratory volume in 1s, *FVC* Forced vital capacity

Severe bronchial asthma is defined as a comorbid asthma diagnosis (International Classification of Disease; ICD J45.9) uncontrolled despite treatment according to Global Initiative on Asthma Step 4 (inhaled steroids combined with long-acting beta l^− 2^-agonists, leukotriene receptor antagonists, or tiotropium) or controlled using Step 5 treatment (Step 4 + oral steroids, anti-IgE, anti-IL5, or temperature-controlled laminar airflow). Asthma is regarded as uncontrolled in cases of nocturnal waking or need for short-acting beta-2 agonists at least twice daily during the past week, exacerbation requiring an oral steroid course during the previous 6 months, or an Asthma Control Test score < 20. The exclusion criteria of heart failure include both systolic and diastolic left ventricular heart failure, but not pulmonary hypertension with right ventricular involvement due to COPD.

### Procedure

**Fig. 1:**
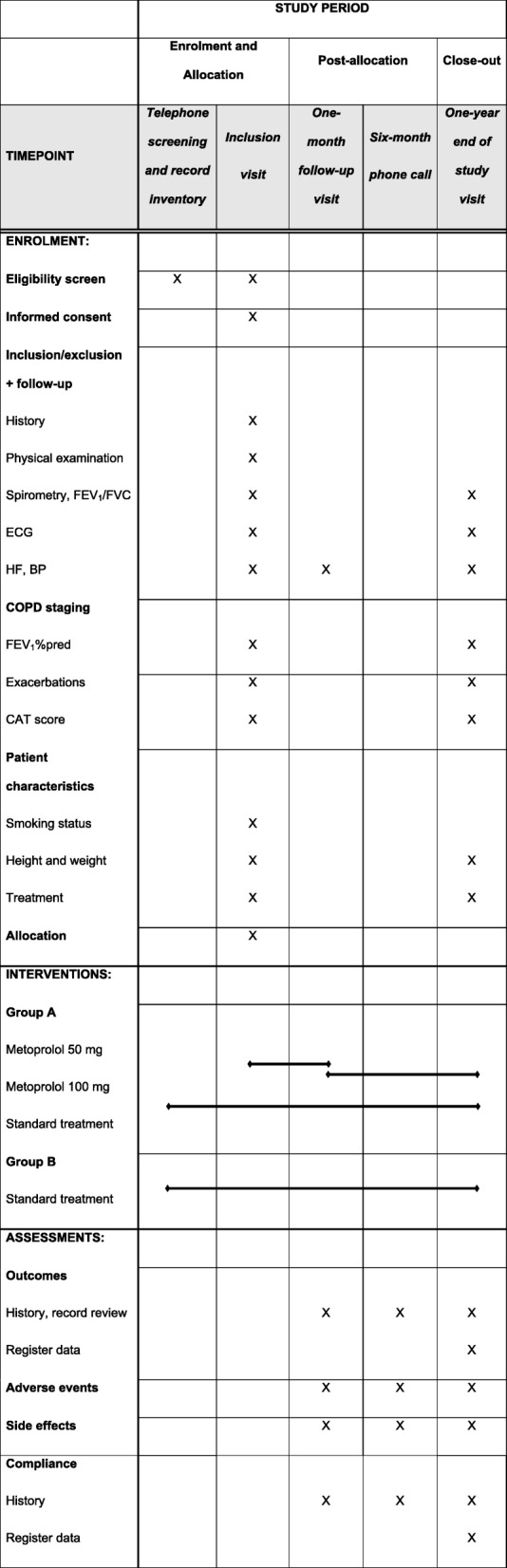
SPIRIT Figure of the trial procedure, including enrolment, data collection, interventions and assessments. COPD staging is performed as GOLD stage I–IV based on FEV_1_%pred and GOLD group A-D based on exacerbation frequency and CAT score [25]. Maintenance treatment includes COPD and cardiovascular treatment in the following groups: Long-acting muscarinic antagonists; long-acting beta-2-agonists; inhaled corticosteroids; roflumilast; long-term oxygen therapy; other COPD treatment, including azithromycin and teofyllamin; ACE/AII-inhibitors; calcium-inhibitors; diuretics; and class I–IV anti-arrhythmics. *BP* blood pressure, *CAT* COPD Assessment Test, *COPD* chronic obstructive pulmonary disease, *ECG* electrocardiography, *FEV*_*1*_ forced expiratory volume in 1 s, *GOLD* Global initiative for Obstructive Lung Disease, *HR* heart rate

Patients who have provided written consent are registered in Smart-Trial (MEDEI ApS, Aalborg, Denmark), a combined password-protected web-based randomization module and electronic case record form (eCRF). Patients who fulfil all inclusion criteria and no exclusion criteria are randomized 1:1 to either standard COPD care + metoprolol or to standard COPD care only. The initial dose of metoprolol is 50 mg, with the aim of increasing to 100 mg at 1 month. If necessary, titration in smaller increments, or reductions in dose, are allowed. Reasons for not titrating to 100 mg could be sinus bradycardia < 50/min, systolic blood pressure < 90 mmHg, or intolerable side effects. The target dose was chosen based on the recommendation for treatment of palpitations and hypertension and because the purpose is to lower the resting heart rate [23]. The medication is prescribed through the standard electronic health record system, with costs covered by the project. Initiation (whether the prescribed drug is dispensed), compliance (proportion of prescribed tablets that are dispensed), and persistence (time on treatment) will be followed via the Swedish Prescribed Drugs Register [24]. The 1-month titration visit is followed by a phone call at 6 months to collect data of compliance and outcomes and by an end-of-study visit at 12 months. The regimen for enrolment, data collection, interventions, and assessment of the trial is shown in Fig. 1. All patients will be assigned an identifying number, recorded in the eCRF together with collected data. A code list connecting patient study numbers with individual Swedish resident personal identification numbers will be kept separately and secured at the participating clinical centers. At completion of collection, a database with data from the eCRF and national registers will be established. The requested SPIRIT check list of an RCT is available as Additional file 2.

### Outcome measures

Mortality data will be obtained from the Swedish Cause of Death Register [26] and include all-cause mortality and cause-specific death due to cardiovascular, respiratory, or other disease at one year. Mortality data are expected to be largely complete due to the ability to follow individuals using the Swedish resident unique personal identification number and the compulsory registration of deceased persons in the Cause of Death Register. A COPD exacerbation is defined as ICD hospitalization discharge code J44.0 or J44.1 or as a course of oral steroids prescribed for worsening COPD symptoms. Cardiovascular events will be defined as recorded ICD codes I20 (angina pectoris); I21 or I22 (myocardial infarction); I48 (atrial fibrillation or flutter); I50.1 to 150.9 (left-sided heart failure); or I61, I63, or G45.9 [(cerebral hemorrhage, stroke, or transient ischemic attack (TIA)]. Data of exacerbations and cardiovascular events will be obtained from history taken at follow-up visits, tracked by review of electronic primary and secondary care records at follow-up visits, and by information collected from the Swedish National Patient Register [27]. All endpoints will be adjudicated by an independent adjudication committee. The adjudication will be blinded, as only the parts of the records describing the actual outcomes will be available to the committee.

### Sample size

Sample size is calculated on the basis of (1) an estimated overall 1-year risk of death of 1% in this mixed cohort of patients at various stages of COPD; (2) a 1-year risk of COPD exacerbations of 20% in a mixed population of primary and secondary care patients; and (3) a 1-year risk of myocardial infarction, atrial fibrillation, other arrhythmias, heart failure, stroke, TIA, or cerebral hemorrhage leading to hospitalization of 2%. The combined 1-year primary composite endpoint risk is estimated as 23% (expected survival probability of 0.77) for individuals randomized to standard care, with an expected variation of 15 to 30% among centers. With a 5% two-sided alpha level and statistical power of 80%, 800 subjects in each group (total *n* = 1600) are required to detect a 25% difference of the primary endpoint in the beta-blocker group from standard treatment only, corresponding to an odds ratio of 0.698 [Cochran Mantel-Haenszel test using nQuery Advisor (Boston, MA, USA)]. In order to compensate for dropouts and crossover between groups, 1700 patients will be included.

### Statistical analysis

The analysis will be conducted according to the intention-to-treat principle with per protocol as a secondary analysis. Differences between studied groups regarding patient baseline characteristics will be evaluated with a Chi-squared test or Fisher’s exact test, as appropriate, for categorical data, a Chi-squared test of trends for stage-of-disease, and an unpaired *t* test for continuous data. Differences in the 1-year composite primary endpoint between groups will be assessed using a log-rank test stratified by treatment center and further evaluated with Cox regression adjusted for center and prognostic factors such as exacerbation frequency, COPD Assessment Test (CAT) score [28], and forced expiratory volume in one second as percentage of predicted value (FEV_1_%pred). The sample size is calculated to have sufficient power to answer the primary research question of the composite measure of total mortality, cardiovascular events, and exacerbations. The same statistical methods, or competing risk survival regression, will be used to evaluate secondary outcomes, using Bonferroni–Holm correction for multiple testing with the aim of describing the relative importance of the components of the primary outcome. The proportional hazard assumption will be evaluated with the *estat phtest* in STATA, which assesses whether the log hazard ratio function is constant over time. A stratified Cox model or time-varying covariates will be used if the proportionality assumption fails.

At a maximum of 3 months following inclusion of the first 400; 800 and respectively 1200 patients, an independent safety monitoring board will review study endpoints in an interim analysis. Variables to be assessed are all-cause death, exacerbation of COPD, and cardiovascular events according to the protocol. Premature termination of the study will be mandated if there is a difference between treatment strategies with significance at the 0.001 alpha level for the primary composite outcome of all-cause mortality, COPD exacerbations, or cardiovascular events.

### Administration of the trial

The steering committee, consisting of national primary investigator Josefin Sundh and sponsor Ole Fröbert of the department of Cardiology and Respiratory Medicine at the Örebro University Hospital, is responsible for the planning and performance of the study. Study administration, coordination, and monitoring are performed by project leaders at the Clinical Research Centre, Örebro University Hospital, Sweden. The BRONCHIOLE trial group includes local primary investigators at several sites (Additional file 1). At the time of the study protocol submission, inclusion is ongoing or planned at 21 sites. Further sites may be added during the study period.

### Data monitoring

In accordance with the principles of the International Conference on Harmonization-Good Clinical Practice, study monitoring will be arranged by the sponsor and occur on-site before, during, and after the trial. The majority of monitoring will be centralized and consist of regular checks of the quality of data in the database. Monitors at the participating centers will review source documents for verification of consistency with the study data recorded in the eCRF.

### Adverse events

An adverse event (AE) is any untoward medical occurrence that does not necessarily have a causal relationship with the treatment. Serious adverse events (SAE) are usually defined as any untoward medical occurrence that results in death, is life-threatening, requires hospitalization or prolongation of existing inpatient hospitalization, results in persistent or significant disability or incapacity, or represents another important medical event. In this trial, medical events are not categorized as AEs or SAEs if they are defined as study endpoints, if they are expected side effects of beta-blocker treatment, or if they are symptoms of an existing disease or an exacerbation of that disease. All AEs will be classified as mild (awareness of sign or symptom, but easily tolerated and not interfering with daily activities), moderate (discomfort of a degree to cause interference with daily activities), or severe (inability to perform normal daily activities).

## Discussion

The BRONCHIOLE study investigates the effect of a commonly used drug for a new indication, metoprolol for COPD without comorbid cardiovascular disease, which could improve important patient-related outcomes. COPD is an irreversible disease with high mortality risk and high health and economic costs [2, 29]. It is the third leading cause of death worldwide [30], and both cardiovascular comorbidity and exacerbations are associated with a high risk of excess mortality [31, 32].

Treatment with beta-blockers for heart failure and following myocardial infarction is evidence-based [5, 6], but underused in patients with COPD [33–35]. As multiple observational studies indicate a general benefit of beta-blockers on important patient-related outcomes in COPD [8–10], it is important to investigate their effects in patients with COPD but exhibiting no overt heart disease in a randomized clinical trial. Previously reported observations of benefits with respect to exacerbations and mortality in COPD could be explained by residual confounding, but there are also several potential hypothetical rationales for a true beneficial effect [13–15, 17–19]. Several authors have pointed to the existing knowledge gap of whether beta-blockers are useful in all patients with COPD, suggesting an urgent need for randomized controlled trials with this aim [14, 36, 37]. Beta-blocker therapy has the potential to be highly cost-effective, and considerable health care and societal benefits could be anticipated if shown effective in patients with COPD without cardiovascular disease.

Bhatt and colleagues are conducting a placebo-controlled, double-blind trial investigating the effect of beta-blockers in COPD without heart disease with COPD exacerbations as the primary outcome [38]. Devereux and colleagues have registered a corresponding placebo-controlled double-blind trial (ISRCTN10497306) investigating bisoprolol in COPD without heart disease, also with COPD exacerbations the primary outcome. Both trials specify a 12-month intervention period with planned enrolment of 1028 and 1574, respectively, compared with 1700 participants in BRONCHIOLE. The studies are complementary to our trial, as they are placebo-controlled, while BRONCHIOLE has a pragmatic real-life design. Evidence from more than one randomized trial is required to bring a change in existing guidelines, and results of the three trials could be instrumental in this respect.

We chose to use a pragmatic design, consistent with recent calls for pragmatic trials designed to show the practical effectiveness of interventions in broad patient groups [20, 21]. We expect our design to facilitate rapid inclusion, but, more importantly, to reflect a “real-world” study population with high external validity and generalizability. In RCTs with highly selected populations, beneficial effects may be overestimated and harm potentially underestimated [20, 21]. Previous pragmatic studies have succeeded in including older and more multi-morbid patients than corresponding ordinary phase IV trials [39]. Despite the fact that most observational studies have shown a positive effect of beta-blockers in COPD, one registry study investigating severe COPD with secondary hypoxemia reported increased mortality in patients using beta-blockers [40]. This may be due to confounding, as subjects on beta-blockers may have had more extensive cardiovascular comorbidity, but these results emphasize the importance of including patients experiencing all stages of COPD, including severe disease, in our trial.

The major potential limitation of our pragmatic design is that treatment is un-blinded. However, in order to ensure objectivity, a central adjudication committee will assess the reported outcomes, in a blinded way. The majority of outcomes will also be confirmed using registry data with expected complete follow-up, including all deaths as well as all hospitalizations and outpatient visits in secondary care due to COPD exacerbations and cardiovascular events. Other potential limitations are that we cannot be certain if the follow-up period is of adequate length or the maximum dose of metoprolol is sufficient.

The exclusion criteria include both absolute and relative indications for beta-blockers. The reason for this choice was a request from the ethical review board to prevent the possibility of withholding beta-blockers from patients in the control group for whom this treatment is guideline indicated.

## Current trial status

The trial was registered on ClinicalTrials.gov on June 25, 2018 (ID: NCT03566667). A pilot phase including 100 patients, initiated July 2018, has been completed in Region Örebro County. The aim of the pilot phase was to identify problems in the design and logistics of the study, and it resulted in minor adjustments in, and clarifications of, the study protocol, such as defining heart failure as left sided systolic or diastolic failure and including exposure to smoke, dust, or gas as inclusion criteria. In October 2018, inclusion started at the first external site. At the time of resubmission, 555 patients have been included at 15 centers. The remaining centers are prepared to begin or are awaiting approval for an amendment from the Ethical Review Board. Inclusion is planned to continue during 2019 and 2020, with follow-up through December 2021. The current protocol version is 1.7, dated 1st of June 2019. A new version 1.8 where the exact number of planned interim analyses are stated, is under review by the Ethical Board and the Medical Products Agency.

## Supplementary information

**Additional file 1.** Participating centers.

**Additional file 2.** SPIRIT Checklist.

## Data Availability

Data cannot be made freely available as they are subject to privacy in accordance with the Swedish Public Access to Information and Secrecy Act but can be provided to researchers upon request, subject to a review of privacy. Requests for data can be sent to the corresponding author.

## Abbreviations

AE: Adverse event
COPD: Chronic obstructive pulmonary disease
CVD: Cardiovascular disease
ECRF: Electronic Case Report Form
FEV_1_: Forced expiratory volume in 1 s
FVC: Forced vital capacity
GOLD: Global Initiative on Obstructive Lung Disease
HF: Heart failure
HR: Heart rate
MI: Myocardial infarction
RCT: Randomized clinical trial
SAE: Serious adverse event
TIA: Transient ischemic attack
