# Supplementary material for: Beta-blockeRs tO patieNts with CHronIc Obstructive puLmonary diseasE (BRONCHIOLE) – Study protocol from a randomized controlled trial
Source: Trials. 2020 Jan 30;21:123. doi: 10.1186/s13063-019-3907-1 (PMC6993405; doi:10.1186/s13063-019-3907-1)
Supplement: Supplementary file 1 — Additional file 1. Participating centers. [file 13063_2019_3907_MOESM1_ESM.docx]

**Additional File 1. Participating centres**

|  | **Ongoing inclusion** | **Planned inclusion** |
| --- | --- | --- |
| Department of Medicine, Sunderbyn Hospital, Luleå |  | x |
| Department of Medicine, Division of Respiratory Medicine and Allergy, University Hospital, Umeå, Sweden | x |  |
| Obackakliniken, Härnösand | x |  |
| Department of Medicine, Gävle Hospital, Gävle | x |  |
| Department of Medicine, Falun Hospital, Falun | x |  |
| Department of Respiratory Medicine, Uppsala University Hospital, Uppsala |  | x |
| Karolinska Trial Alliance, Stockholm | x |  |
| Department of Internal Medicine, Central Hospital, Karlstad | x |  |
| Academic primary health care centre of Kil | x |  |
| Academic primary health care centre of Skoghall | x |  |
| Department of Primary Care, Tunafors Family Center, Eskilstuna | x | x |
| The Centrum Primary Health Care Center, Flen |  | x |
| Department of Respiratory Medicine, Örebro University Hospital, and Research Clinical Unit, Örebro | x |  |
| Primary Care Trial Centre, Skövde | x |  |
| Angered Hospital, Göteborg |  | x |
| COPD center, Sahlgrenska University Hospital, Institute of Medicine, University of Göteborg, Göteborg | x |  |
| Carlanderska Hospital, Göteborg | x |  |
| Primary Care Trial Centre, Göteborg | x |  |
| Clinical Trial Consultants, Linköping | x | x |
| BTH Research and Education Clinic, Karlskrona | x |  |
| Department of Respiratory Medicine, Vejle, Denmark |  | x |
