# Supplementary material for: Beta-blockeRs tO patieNts with CHronIc Obstructive puLmonary diseasE (BRONCHIOLE) – Study protocol from a randomized controlled trial
Source: Trials. 2020 Jan 30;21:123. doi: 10.1186/s13063-019-3907-1 (PMC6993405; doi:10.1186/s13063-019-3907-1)
Supplement: Supplementary file 2 — Additional file 2. SPIRIT Checklist. [file 13063_2019_3907_MOESM2_ESM.docx]

Additional File 2. SPIRIT Checklist

| Section/item | Item No | Description | Addressed on page number |
| --- | --- | --- | --- |
| **Administrative information** | | |  |
| Title | 1 | Descriptive title identifying the study design, population, interventions, and, if applicable, trial acronym | Study protocol: Page 1  Study protocol paper: Page 1 |
| Trial registration | 2a | Trial identifier and registry name. If not yet registered, name of intended registry | Study protocol paper: Page 2 |
|  | 2b | All items from the World Health Organization Trial Registration Data Set |  |
| Protocol version | 3 | Date and version identifier | Study protocol:  Page 1 + side heading |
| Funding | 4 | Sources and types of financial, material, and other support | Study protocol paper: Page 15 |
| Roles and responsibilities | 5a | Names, affiliations, and roles of protocol contributors | Study protocol: Page 1  Study protocol paper: Page 1 + 15 |
|  | 5b | Name and contact information for the trial sponsor | Study protocol: Page 1  Study protocol paper: Page 1 |
|  | 5c | Role of study sponsor and funders, if any, in study design; collection, management, analysis, and interpretation of data; writing of the report; and the decision to submit the report for publication, including whether they will have ultimate authority over any of these activities | Study protocol: Page 15 + 20  Study protocol paper: Page 11 + 16 |
|  | 5d | Composition, roles, and responsibilities of the coordinating centre, steering committee, endpoint adjudication committee, data management team, and other individuals or groups overseeing the trial, if applicable (see Item 21a for data monitoring committee) | Study protocol: Page 13 +15 + 20  Study protocol paper: Page 9-11 |
| Introduction |  |  |  |
| Background and rationale | 6a | Description of research question and justification for undertaking the trial, including summary of relevant studies (published and unpublished) examining benefits and harms for each intervention | Study protocol: Page 5-8  Study protocol paper: Page 3-4 |
|  | 6b | Explanation for choice of comparators | Study protocol: Page 5-8  Study protocol paper: Page 3-4 |
| Objectives | 7 | Specific objectives or hypotheses | Study protocol: Page 8  Study protocol paper: 5-6 |
| Trial design | 8 | Description of trial design including type of trial (e.g., parallel group, crossover, factorial, single group), allocation ratio, and framework (e.g., superiority, equivalence, noninferiority, exploratory) | Study protocol: Page 9-11  Study protocol paper: 4-5 |
| Methods: Participants, interventions, and outcomes | | |  |
| Study setting | 9 | Description of study settings (e.g., community clinic, academic hospital) and list of countries where data will be collected. Reference to where list of study sites can be obtained | Study protocol: Page 9  Study protocol paper: 11 + 22 |
| Eligibility criteria | 10 | Inclusion and exclusion criteria for participants. If applicable, eligibility criteria for study centres and individuals who will perform the interventions (e.g., surgeons, psychotherapists) | Study protocol: page 3 + 9-10  Study protocol paper page 6-7 |
| Interventions | 11a | Interventions for each group with sufficient detail to allow replication, including how and when they will be administered | Study protocol: page 11  Study protocol paper page 8-9 + 20-22 |
|  | 11b | Criteria for discontinuing or modifying allocated interventions for a given trial participant (e.g., drug dose change in response to harms, participant request, or improving/worsening disease) | Study protocol: page 11  Study protocol paper page 8-9 |
|  | 11c | Strategies to improve adherence to intervention protocols, and any procedures for monitoring adherence (e.g., drug tablet return, laboratory tests) | Study protocol: page 11  Study protocol paper page 8-9 |
|  | 11d | Relevant concomitant care and interventions that are permitted or prohibited during the trial | Study protocol: page 11  Study protocol paper page 7-8 |
| Outcomes | 12 | Primary, secondary, and other outcomes, including the specific measurement variable (e.g., systolic blood pressure), analysis metric (e.g., change from baseline, final value, time to event), method of aggregation (e.g., median, proportion), and time point for each outcome. Explanation of the clinical relevance of chosen efficacy and harm outcomes is strongly recommended | Study protocol: page 12  Study protocol paper page 9 + 20-22 |
| Participant timeline | 13 | Time schedule of enrolment, interventions (including any run-ins and washouts), assessments, and visits for participants. A schematic diagram is highly recommended (see Figure) | Study protocol: page 10-11  Study protocol paper: page 8-9 + 20-22 |
| Sample size | 14 | Estimated number of participants needed to achieve study objectives and how it was determined, including clinical and statistical assumptions supporting any sample size calculations | Study protocol: page 14  Study protocol paper page 9-10 |
| Recruitment | 15 | Strategies for achieving adequate participant enrolment to reach target sample size | Study protocol: page 9  Study protocol paper page 6 |
| **Methods: Assignment of interventions (for controlled trials)** | | |  |
| Allocation: |  |  |  |
| Sequence generation | 16a | Method of generating the allocation sequence (e.g., computer-generated random numbers), and list of any factors for stratification. To reduce predictability of a random sequence, details of any planned restriction (e.g., blocking) should be provided in a separate document that is unavailable to those who enrol participants or assign interventions | Study protocol: page 11  Study protocol paper page 8 |
| Allocation concealment mechanism | 16b | Mechanism of implementing the allocation sequence (e.g., central telephone; sequentially numbered, opaque, sealed envelopes), describing any steps to conceal the sequence until interventions are assigned | Study protocol: page 11  Study protocol paper page 8 |
| Implementation | 16c | Who will generate the allocation sequence, who will enrol participants, and who will assign participants to interventions | Study protocol: page 11  Study protocol paper page 8 |
| Blinding (masking) | 17a | Who will be blinded after assignment to interventions (e.g., trial participants, care providers, outcome assessors, data analysts), and how | 9 |
|  | 17b | If blinded, circumstances under which unblinding is permissible, and procedure for revealing a participant’s allocated intervention during the trial | N/A |
| **Methods: Data collection, management, and analysis** | | |  |
| Data collection methods | 18a | Plans for assessment and collection of outcome, baseline, and other trial data, including any related processes to promote data quality (e.g., duplicate measurements, training of assessors) and a description of study instruments (e.g., questionnaires, laboratory tests) along with their reliability and validity, if known. Reference to where data collection forms can be found, if not in the protocol | Study protocol: page 10-11  Study protocol paper: Page 20-22 |
|  | 18b | Plans to promote participant retention and complete follow-up, including list of any outcome data to be collected for participants who discontinue or deviate from intervention protocols | Study protocol: page 10-11  Study protocol paper: Page 20-22 |
| Data management | 19 | Plans for data entry, coding, security, and storage, including any related processes to promote data quality (e.g., double data entry; range checks for data values). Reference to where details of data management procedures can be found, if not in the protocol | Study protocol: page 14  Study protocol paper: Page 8-9 |
| Statistical methods | 20a | Statistical methods for analysing primary and secondary outcomes. Reference to where other details of the statistical analysis plan can be found, if not in the protocol | Study protocol: page 12-13  Study protocol paper page 10-11 |
|  | 20b | Methods for any additional analyses (e.g., subgroup and adjusted analyses) | Study protocol: page 12-13  Study protocol paper page 10-11 |
|  | 20c | Definition of analysis population relating to protocol non-adherence (e.g., as randomized analysis), and any statistical methods to handle missing data (e.g., multiple imputation) | Study protocol: page 12-13  Study protocol paper page 10 |
| **Methods: Monitoring** | | |  |
| Data monitoring | 21a | Composition of data monitoring committee (DMC); summary of its role and reporting structure; statement of whether it is independent from the sponsor and competing interests; and reference to where further details about its charter can be found, if not in the protocol. Alternatively, an explanation of why a DMC is not needed | Study protocol: Page 14  Study protocol paper: Page 9 + 11-12 |
|  | 21b | Description of any interim analyses and stopping guidelines, including who will have access to these interim results and make the final decision to terminate the trial | Study protocol: Page 13  Study protocol paper: Page 10-11 |
| Harms | 22 | Plans for collecting, assessing, reporting, and managing solicited and spontaneously reported adverse events and other unintended effects of trial interventions or trial conduct | Study protocol: Page 16-18  Study protocol paper: Page 10-11 |
| Auditing | 23 | Frequency and procedures for auditing trial conduct, if any, and whether the process will be independent from investigators and the sponsor | Study protocol: Page 14  Study protocol paper: Page 11 |
| Ethics and dissemination | | |  |
| Research ethics approval | 24 | Plans for seeking research ethics committee/institutional review board (REC/IRB) approval | Study protocol: Page 15  Study protocol paper: Page 16 |
| Protocol amendments | 25 | Plans for communicating important protocol modifications (e.g., changes to eligibility criteria, outcomes, analyses) to relevant parties (e.g., investigators, REC/IRBs, trial participants, trial registries, journals, regulators) | Study protocol: Page 13 |
| Consent or assent | 26a | Who will obtain informed consent or assent from potential trial participants or authorized surrogates, and how (see Item 32) | Study protocol: Page 15-16  Study protocol paper: Page 5 |
|  | 26b | Additional consent provisions for collection and use of participant data and biological specimens in ancillary studies, if applicable | N/A |
| Confidentiality | 27 | How personal information about potential and enrolled participants will be collected, shared, and maintained in order to protect confidentiality before, during, and after the trial | Study protocol: Page 14  Study protocol paper: Page 8-9 |
| Declaration of interests | 28 | Financial and other competing interests for principal investigators for the overall trial and each study site | Study protocol: Page 15  Study protocol paper: Page 15 |
| Access to data | 29 | Statement of who will have access to the final trial dataset, and disclosure of contractual agreements that limit such access for investigators | Study protocol: Page 14 + 20  Study protocol paper: Page 9 |
| Ancillary and post-trial care | 30 | Provisions, if any, for ancillary and post-trial care, and for compensation to those who suffer harm from trial participation | Study protocol: Page 15 |
| Dissemination policy | 31a | Plans for investigators and sponsor to communicate trial results to participants, healthcare professionals, the public, and other relevant groups (e.g., via publication, reporting in results databases, or other data sharing arrangements), including any publication restrictions | Study protocol: Page 19-20 |
|  | 31b | Authorship eligibility guidelines and any intended use of professional writers | N/A |
|  | 31c | Plans, if any, for granting public access to the full protocol, participant-level dataset, and statistical code | Study protocol paper: Page 16 |
| Appendices |  |  |  |
| Informed consent materials | 32 | Model consent form and other related documentation given to participants and authorized surrogates | N/A |
| Biological specimens | 33 | Plans for collection, laboratory evaluation, and storage of biological specimens for genetic or molecular analysis in the current trial and for future use in ancillary studies, if applicable | N/A |
